# Testis Scintigraphy in a Patient with Acute Lymphoblastic Leukemia

**DOI:** 10.4274/Mirt.314

**Published:** 2014-02-05

**Authors:** Mine Şencan Eren, Murat Koç, Hale Ören, Şermin Özkal, Hatice Durak

**Affiliations:** 1 Dokuz Eylül University School of Medicine, Department of Nuclear Medicine, İzmir, Turkey; 2 Dokuz Eylül University School of Medicine, Department of Pediatric Hematology, İzmir, Turkey; 3 Dokuz Eylül University School of Medicine, Department of Pathology, İzmir, Turkey

**Keywords:** lymphoblastic leukemia, acute, Spermatic cord torsion, scintigraphy

## Abstract

Acute lymphoblastic leukemia (ALL) is a pediatric malignancy associated with remissions and relapses. Common relapsing sitesare meninges, testis and ovary. Testicular scintigraphy is a highly specific modality used mainly in the differential diagnosis of testicular torsion and epidydimitis/epidydimo-orchitis. There is only one interesting image on leukemic infiltration with scrotal scintigraphy in the literature. The aim of this case presentation is to report that although the scintigraphic appearance of testicular torsion was observed in a patient with the diagnosis of ALL, testicular ALL infiltration was revealed in pathologic examination.

**Conflict of interest:**None declared.

## Abstract (tr)

Akut lenfoblastik lösemi (ALL), çocukluk çağında görülen, relaps ve remisyonlarla seyreden bir malignitedir. Relapsları sıklıkla meninksler, testis ve overlerde izlenir. Testis sintigrafisi, esas olarak epididimit/epididimoorşit ve testis torsiyonu ayırıcı tanısında kullanılan oldukça spesifik bir yöntemdir. Literatürde skrotal sintigrafide lösemik infiltrasyon ile ilgili sadece bir adet görüntü mevcuttur. Bu olgu sunumunda amacımız, ALL tanısı ile takip edilen hastada sintigrafik olarak testis torsiyonu görünümü olmasına rağmen, patolojik incelemede testiste ALL infiltrasyonu görüldüğünü bildirmektir.

## INTRODUCTION

Scrotal mass lesions may present with or without pain. Testicular and extra-testicular painless non-tumoral lesions are benign pathologies such as varicocele and spermatocele. Those pathologies account for more than half of the scrotal pathologies in pediatric age. Diagnosis of these diseases is made with physical examination and simple radiological imaging studies. Painful testicular mass lesions may result from many various causes ranging from torsion of spermatic cord to trauma and intra-tumoral hemorrhage (1,2).

Acute lymphoblastic leukemia (ALL) accounts for approximately 30% of pediatric tumors (3,4). In ALL, around 5-8 percent of extra-medullar relapses are in the testis (5). The rate of testicular infiltration is reported to be around 62% in ALL (6).

Ultrasonography is among the first-line diagnostic methods in scrotal lesions associated with pain. Moreover, testicular scintigraphy and sometimes magnetic resonance imaging (MRI) can be used in differential diagnosis of testicular torsion, epidydimitis, abscess and tumoral lesions in case of clinical necessity.

In this current case report, we are presenting a patient with ALL who experienced severe pain in his testis. He had a scintigraphic appearance of testicular torsion, but histopathology revealed testicular ALL infiltration.

## CASE REPORT

A 14-year old male patient was followed up with the diagnosis of ALL since 2007 and had bone marrow transplantation after central nervous system (CNS) and bone marrow relapse. He was referred to pediatric emergency department because of severe pain in his left testis for 2 days.

In the physical examination of the patient, diffuse rashes throughout the body and diffuse tenderness in abdominal supra-pubic region were observed. He also had abdominal pain and diarrhea. Laboratory values were Hgb:12.9 g/dL, WBC:7.3x10^3^ μL, PLT:185x10^3^ μL, Na:133 mmol/L, K:2,6 mmol/L, Ca:5.4 mg/dL and Mg:0.3 mg/dL and significant electrolyte imbalance was found. Fluid replacement therapy was started in the emergency department. Clinically, cutaneous and gastrointestinal graft-versus-host-disease (GVHD) and testis involvement/testis torsion were considered. 

US examination was performed for the pain in his left testis. Right testis was 28x26 mm in size and parenchymal echogenity was normal. Left testis was 35x30 mm with decreased and heterogeneous parenchymal echogenity. Increased vascularity was observed in structures surrounding the left testis and at the left epidydimis. Radiological diagnoses of ALL infiltration and/or inflammation-epidydimo-orchitis in left testis were considered, but definite diagnosis could not be made.

The patient was referred to the nuclear medicine department to have a testicular scintigraphy in order to eliminate testicular torsion.

In the physical examination of the patient in nuclear medicine department, apparent swelling and hyperemia were observed in the left testis. Palpation was associated with severe pain and rigidity was noted in left testis.

**Figure 1:**
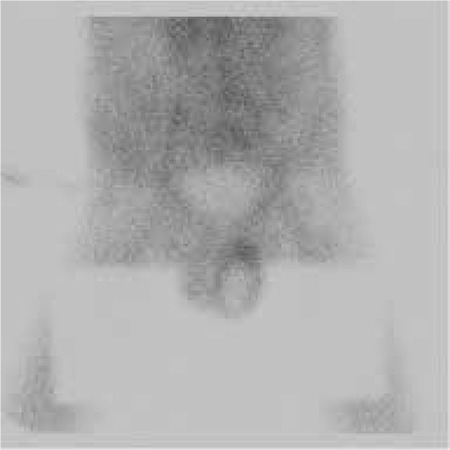
The anterior static view of testis scintigraphy showing hypoactive left testicular area surrounded by an hyperactive halo.

**Figure 2:**
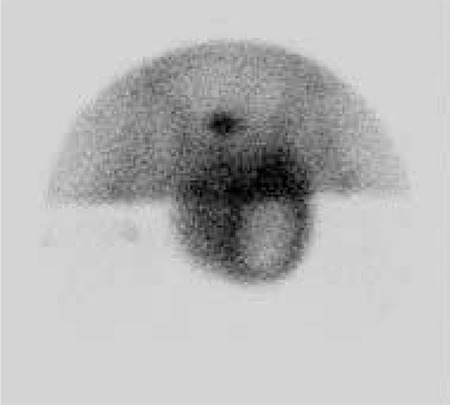
The anterior pinhole view of testis scintigraphy showing hypoactive left testicular area surrounded by an hyperactive halo.

**Figure 3:**
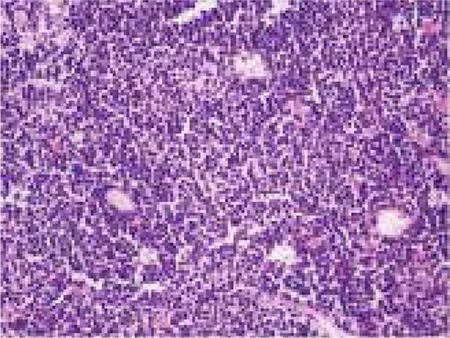
The tumor cells are observed in violet by hematoxilen-eosine staining.

**Figure 4:**
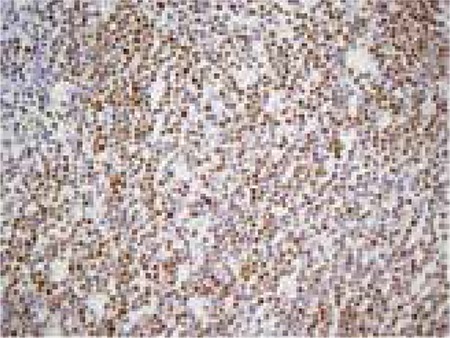
The tumor cells are observed brown in nucleus by Terminal deoxynucleotidyl transferase (TDT) immunohistochemical staining.

**Figure 5:**
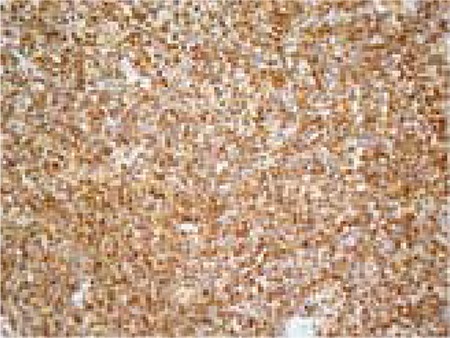
The tumor cells are observed brown in cytoplasm by CD10 immunohistochemical staining.

Penis was elevated upwards and fixed to pelvic bone with adhesive band. Imaging was performed with Low Energy General Purpose (LEGP) collimator. Following intravenous administration of 10 mCi Tc-99m pertechnetate, dynamic perfusion images were obtained in order to examine blood supply of testis, followed by pinhole and static images. In dynamic images, perfusion in the area suggestive of left testis was decreased. In static images, the left testicular area was hypoactive and a hyperactive halo surrounding this area was noted (Figure 1). Those findings were observed better in pinhole images (Figure 2). In the light of those findings, patient was considered to have subacute left testicular torsion. Patient underwent left orchidectomy. In the histopathological examination, lymphoblastic lymphoma infiltration was observed in testicular tissue. Diffuse lymphoblastic cells in the testicular tissue had replaced the entire testicular structure (Figure 3). Immunohistochemical staining revealed Terminal Deoxynucleotidyl Transferase (TDT) (Figure 4) and CD-10 (Figure 5) positivity in lymphoblastic cells.

## LITERATURE REVIEW AND DISCUSSION

Testicular leukemic infiltration is diagnosed with the presence of leukemic cells in testicular interstitial tissue. Wakasa et al. histopathologically demonstrated that leukemic infiltration can be diffuse or in the form of patches in perivascular area and interstitium of the testis (7). In their study, presence of leukemic cells was shown in lumen of capillary structures and inside testicular lymphatics. Leukemic cells may access intra-luminal site by migrating from interstitium into lumen of vessel (7). In a study conducted on lymphoma and leukemia patients with testicular infiltration, Mazzu et al. observed enlargement in involved testis in all cases in US (8). Moreover, they found diffuse and focal tumor infiltration in testis in US. Increased blood flow was observed in the lesion while echogenity was decreased. In the light of the findings obtained from US, they demonstrated that testicular infiltration of round-cell tumors and infection could not be differentiated (8). Aso et al. also observed increased blood flow similar to the inflammatory process secondary to testicular infiltration (9).

Calama reported in a series of 653 cases aged 0-18 years that testicular hematopoiesis could be at the rate of 26.5 percent (10). In the presence of serious conditions such as infection, hypoxemia and blood diseases, extra-medullary hematopoiesis in testis is induced (10). Testis is a “sanctuary organ” since there is a blood-gonad barrier. It is specified in the literature that passage of chemotherapeutic agents into testicular tissue are inhibited by blood-testis barrier. This reason provides support to clarify underlying cause of leukemic infiltration in the testis (8). Moreover, it is also known that relapse is common in ALL patients with non-treated or minimal residual disease.

Testicular torsion is among emergencies of nuclear medicine and this condition is not rare. Occlusion occurs in vascular structures supplying blood to the testis when spermatic cord twists, resulting in testicular ischemia.

Testicular torsion can be differentiated from other testicular pathologies using testicular scintigraphy at the rate of 90 percent (11). In a normal testicular scintigraphy, bilateral and mildly intense radiopharmaceutical uptake is observed. Sometimes, a minimal asymmetry may be present (12,13). Testicular torsion and other pathologies may have totally different appearances (12,13). If torsion is present in testicular scintigraphy, a photopenic defect is observed since there will be no radiopharmaceutical uptake in the involved area, typically due to the occlusion developed in spermatic cord (12,13,14). Sensitivity and specificity of radionuclide scintigraphy in diagnosing testicular torsion was reported as 98% and 100%, respectively (15). 

Ultrasonography is among the first-line imaging methods in patients under follow-up for scrotal lesions (1,2). Testis may appear normal within first several hours of torsion. Testicular expansion and diffuse hypo-echogenity is observed 4 hours following onset of torsion. Hemorrhage and necrosis-dependent heterogeneously echoic appearance is noted when time elapses. Blood flow to testis with torsion decreases or completely ceases, while blood flow increases in inflammatory processes. Intratesticular flow is not observed in missed torsion, while blood flow increases in peritesticular tissue. Size of testis is small in chronic torsion and it is usually hypo-echoic. Diagnostic sensitivity and specificity of Color Doppler US in testicular torsion was reported as 86 and 100%, respectively (16).

For patients presented with suspected testicular torsion, there are publications recommending that ultrasonography and radionuclide scintigraphy is performed as soon as possible after patient is examined (17).

US may sometimes be inadequate to differentiate vascular diseases, inflammatory process and solid lesions. In such cases, MRI is used. MRI not only evaluates extent of existent disease, but it may also more clearly differentiate solid and cystic lesions and vascular abnormalities (18).

There is only one interesting image on leukemic infiltration with scrotal scintigraphy in the literature. They reported that in scrotal scintigraphy, the appearance of ALL infiltration may be similar to that of epidydimo-orchitis (11).

Since there was occlusion secondary to leukemic infiltration originating from ALL in testicular vessels in the case presented, vascular block has occurred in vessels supplying blood to testis and therefore, decrease in blood supply was observed in testicular scintigraphy.

In this case report, we reported that an appearance resembling testicular torsion may occur as a result of thrombosis in vascular structures due to testicular ALL infiltration, which should be kept in mind while evaluating testicular scintigraphy.
